# Poly(acryloyl hydrazide), a versatile scaffold for the preparation of functional polymers: synthesis and post-polymerisation modification†
†Electronic supplementary information (ESI) available: Additional NMR and UV-Vis spectra, additional tables and proposed mechanism for impurities. See DOI: 10.1039/c7py00535k


**DOI:** 10.1039/c7py00535k

**Published:** 2017-07-03

**Authors:** Daniel N. Crisan, Oliver Creese, Ranadeb Ball, Jose Luis Brioso, Ben Martyn, Javier Montenegro, Francisco Fernandez-Trillo

**Affiliations:** a School of Chemistry , University of Birmingham B15 2TT , UK . Email: f.fernandez-trillo@bham.ac.uk; b School of Chemistry , University of Warwick CV47AL , UK; c Departamento de Química Orgánica y Centro Singular de Investigación en Química Biolóxica e Materiais Moleculares (CIQUS) , Universidade de Santiago de Compostela E-15782 , Spain . Email: javier.montenegro@usc.es

## Abstract

Here we present the synthesis and post-polymerisation modification of poly(acryloyl hydrazide), a versatile scaffold for the preparation of functional polymers: poly(acryloyl hydrazide) was prepared from commercially available starting materials in a three step synthesis on a large scale, in good yields and high purity. Our synthetic approach included the synthesis of a Boc-protected acryloyl hydrazide, the preparation of polymers *via* RAFT polymerisation and the deprotection of the corresponding Boc-protected poly(acryloyl hydrazide). Post-polymerisation modification of poly(acryloyl hydrazide) was then demonstrated using a range of conditions for both hydrophilic and hydrophobic aldehydes. These experiments demonstrate the potential of poly(acryloyl hydrazide) as a scaffold in the synthesis of functional polymers, in particular those applications where *in situ* screening of the activity of the functionalised polymers may be required (*e.g.* biological applications).

## Abstract 2

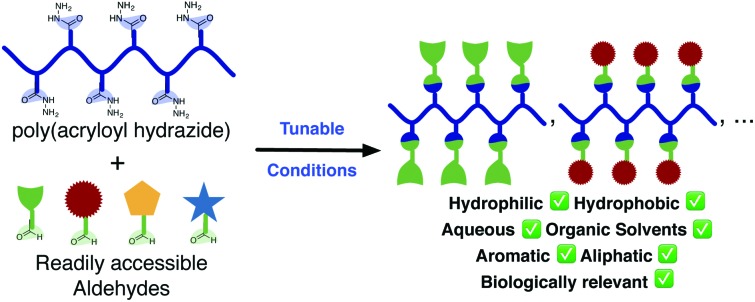
Here we present the synthesis of poly(acryloyl hydrazide), a versatile scaffold for the preparation of functional polymers, and its post-polymerisation modification using a wide range of conditions.

## Introduction

There is an increasing interest in developing polymers for biomedical applications and we now increasingly see polymers that play an “active” role in biology and reproduce or interact with biological functions. Representative examples include glycopolymers that mimic and interfere with glycan recognition,^1^–^4^ polymers for gene delivery that mimic some of the characteristics of viral vectors,^5^–^9^ or therapeutic polymers such as antimicrobial polymers.^10^–^13^ One common requirement when developing polymers for these applications is the need to synthesise libraries of polymers that incorporate highly functional monomers based on for instance carbohydrates, amine or cationic moieties. However, and despite the availability of a large toolbox for the synthesis of polymers,^14^–^17^ there are still many functional groups which are incompatible with existing polymerisation techniques. A common approach to solve this incompatibility is to employ post-polymerisation modification,^18^–^20^ where polymer “scaffolds” are made with reactive moieties that are inert to the polymerisation conditions, but can then be modified post-polymerisation to give other functional groups. The success of this strategy relies on the nearly quantitative conversion of this initial reactive moiety to give functionalised polymers. Not surprisingly, these post-polymerisation protocols have often relied on highly efficient and orthogonal chemistries, *i.e.* click chemistries.^21^–^26^

Despite the progress in this area, one potential limitation of these post-polymerisation strategies is the low aqueous solubility and stability of some of these reactive polymer scaffolds. Thus, additional steps must be employed following post-polymerisation modification and prior to biological evaluation, including the removal of protecting groups. Moreover, the current paradigm assumes that candidate polymers need to be isolated/purified prior to biological evaluation. However, this is inefficient, time-consuming and expensive, since efforts are invested in isolating candidate polymers that do not show any biological activity. The introduction of automation to polymer synthesis has the potential to facilitate some of these steps,^27^ but it can result in even larger libraries of functional polymers, with the subsequent increase in cost associated to purification and isolation.

To address some of these limitations, we have recently reported the application of poly(acryloyl hydrazide) as a reactive polymer scaffold for the *in situ* preparation of polymeric gene vectors for the delivery of siRNA.^27^ Polymers carrying hydrazides as reactive moieties are ideal to develop a post-polymerisation strategy that works in aqueous conditions, and that eliminates purification and isolation steps following post-polymerisation modification. The coupling reaction between hydrazides and aldehydes is orthogonal to many biologically relevant functional groups (*e.g.* hydroxyls, acids or amines) and produces water as a by-product.^28^ Thus, in the absence of interference from the used aldehydes, there is no need to purify candidate polymers after the post-polymerisation reaction. This is often the case for biological applications that benefit from a multivalent effect such as lectin binding.^29^ Also, the formed hydrazone is relatively stable at physiological pH (*i.e.* 5–7),^29^ and the biological activity of the functional polymers can be evaluated without having to reduce the hydrazone.^27^,^30^ Finally, hydrazides are weakly protonated under physiological conditions (p*K*_a_H ∼ 5) and thus poly(hydrazide)s are normally non-toxic.^27^ Despite all of these features, the use of poly(hydrazide)s as a reactive scaffold had been limited to the preparation of glycopolymers,^29^,^31^ and for pH-responsive drug delivery.^32^–^36^ Alternative elegant strategies using poly(alkoxyamine)s^37^,^38^ and poly(aldehyde)s^39^–^41^ have also been explored.

Here, we evaluate the potential of poly(acryloyl hydrazide) as a reactive scaffold for post-polymerisation functionalisation. First, we report the synthesis of poly(acryloyl hydrazide) from a Boc-protected monomer using RAFT polymerisation. Then, we evaluate its functionalisation by reacting with aldehydes and explore a range of conditions. Overall, our results demonstrate that poly(acryloyl hydrazide) is a versatile reactive scaffold that can mediate the synthesis of polymers carrying a wide range of functionalities, including acidic and basic moieties, biologically relevant functionalities, and aliphatic and aromatic side-chains. The efficiency of the hydrazide–aldehyde coupling can be modulated by tuning the reaction conditions, including the use of both aqueous and organic conditions, to yield polymers with a consistent degree of functionalisation.

## Experimental

### Materials

2-((Ethylthio)carbonothioyl)thio-2-methylpropanoic acid (**CTA1**) was synthesised according to protocols described in the literature.^42^ Cyanomethyl methyl(4-pyridyl)carbamodithioate (**CTA2**) was purchased from Sigma-Aldrich® and used without further purification. 2,2′-Azobis[2-(2-imidazolin-2-yl)propane] dihydrochloride (**VA-044**) was purchased from Fluorochem and used without further purification. All other chemicals were purchased from Sigma-Aldrich®, Fisher Scientific®, VWR® or Acros®, and used without further purification. All solvents were Reagent grade or above, purchased from Sigma-Aldrich®, Fisher Scientific® or VWR®, and used without further purification.

Nuclear Magnetic Resonance (NMR) spectra were recorded on either a Bruker Avance III 300 MHz or a Bruker Avance III 400 MHz spectrometer. Chemical shifts are reported in ppm (units) referenced to the following solvent signals: dimethylsulfoxide (DMSO)-*d*_6_ H 2.50 and D_2_O H 4.79. Infrared (IR) spectra were recorded on a PerkinElmer Spectrum Two FT-IR spectrometer. Ultraviolet–visible (UV-vis) spectra were recorded on a Cary 50 Spectrophotometer. Gel Permeation Chromatography (GPC) was performed with a Shimadzu Prominence LC-20A fitted with a Thermo Fisher Refractomax 521 Detector and a SPD20A UV-vis Detector. Boc-Protected poly(acryloyl hydrazide) (**Boc-P_*x*_**) was analysed using 0.05 M LiBr in dimethylformamide (DMF) at 60 °C, or 0.005 M NH_4_BF_4_ in DMF at 50 °C, as the eluent and a flow rate of 1 mL min^–1^. The instrument was fitted with a Polymer Labs PolarGel guard column (50 × 7.5 mm, 5 μm) followed by two PLGel PL1110–6540 columns (300 × 7.5 mm, 5 μm). Molecular weights were calculated based on a standard calibration method using polymethylmethacrylate. Poly(acryloyl hydrazide) **P_*x*_** was analysed using Dulbecco's Phosphate Buffered Saline 0.0095 M (PO_4_) without Ca and Mg as the eluent and a flow rate of 1 mL min^–1^. The instrument was fitted with an Agilent PL aquagel-OH column (300 × 7.5 mm, 8 mm) and run at 35 °C.

Dialysis was carried out in deionised water at room temperature for a minimum of 48 hours using a Spectra/Por 6 1000 Molecular weight cut-off (MWCO) 38 mm width membrane.

### *tert*-Butyl-2-acryloylhydrazine-1-carboxylate (**M1**)

Acrylic acid (3.8 ml, 55.00 mmol) and *tert*-butyl carbazate (8.9 g, 66.0 mmol) were dissolved in a H_2_O : THF mixture (2 : 1, 180 ml) at r.t. *N*-(3-Dimethylaminopropyl)-*N*′-ethylcarbodiimide hydrochloride (11.8 g, 61.3 mmol) was added in portions to the solution over 15 minutes and left stirring for 3 h. The solution was extracted with ethyl acetate (EtOAc) (3 × 75 ml) and the organic layer was washed with 0.1 M HCl (3 × 75 ml), H_2_O (50 ml) and brine (2 × 50 ml). The organic phase was dried with Na_2_SO_4_ and the solvent was removed under reduced pressure to afford the crude product as a white solid. The crude product was purified by recrystallization from EtOAc (70 °C to r.t.) to afford a white crystalline powder (5.1 g, 50% yield): *R*_f_ = 0.87 (100% EtOAc); ^1^H-NMR (300 MHz, DMSO-*d*_6_) *δ* (ppm) 9.79 (s, 1H), 8.84 (s, 1H), 6.17–6.20 (m, 2H), 5.69 (dd, ^3^*J*_*H*,*H*_ = 7.8, 4.5 Hz, 1H), 1.40 (s, 9H). ^13^C-NMR (100 MHz, DMSO-*d*_6_) *δ* (ppm) 164.3 (s), 155.3 (s), 129.4 (d), 126.2 (t), 79.2 (s), 28.1 (q). IR (neat) *ν*_max_ 3311 (m sh, N–H), 3221 (m sh, N–H), 2981 (w sh, C–H), 1715 (s sh, C=O), 1668 (s sh, C=O) cm^–1^.

### Acryloylhydrazide hydrochloride (**M2**)

Hydrazide **M1** (2.0 g, 10.7 mmol) in 1 M HCl_(aq)_ (80 ml) was stirred at 0 °C for 24 h and stirred a further 48 h at r.t. Excess HCl was removed under reduced pressure without heating the solution. Water was removed by lyophilisation to afford a white crystalline powder (0.9 g, 68% yield): ^1^H-NMR (300 MHz, DMSO-*d*_6_) *δ* (ppm) 11.44 (s, 1H), 6.26–6.39 (m, 2H), 5.85 (dd, ^3^*J*_*H*,*H*_ = 9.6, 2.5 Hz, 1H). ^13^C-NMR (100 MHz, DMSO-*d*_6_) *δ* (ppm) 163.9 (s), 129.1 (d), 127.7 (t).

### Poly(*tert*-butyl-2-acryloylhydrazine-1-carboxylate) (**Boc-P_*x*_**)

In a typical experiment, a solution of 4,4′-azobis(4-cyanovaleric acid) (V-501) (18.4 mg, 64.0 μmol) in DMSO (1.5 mL) and a solution of **CTA1** (72.3 mg, 32.2 μmol) in DMSO (1.5 mL) were added sequentially to a solution of *tert*-butyl-2-acryloylhydrazine-1-carboxylate (3.0 g, 16.1 mmol) in DMSO (14.9 mL). A 50 μL aliquot of this solution was taken at this stage to aid in the calculation of conversion. The reaction mixture was then sealed and degassed with Argon for 30 min. The degassed solution was left to react at 70 °C for 7 h. The reaction was stopped by allowing it to cool down to room temperature and by exposing it to air. A 50 μL aliquot of this solution was taken at this stage to aid in the calculation of conversion. The polymer was purified by dialysis against water. The water was removed by lyophilisation and by drying in a desiccator with P_2_O_5_ to afford 2.2 g of **Boc-P_40_** as an off-white powder (73% yield). UV (DMSO) *λ*_max_ 300 nm. ^1^H-NMR (300 MHz, DMSO-*d*_6_) *δ* (ppm) 9.22 (br, 1H, N*H*), 8.60 (br, 1H, N*H*), 2.03 (br, 1H, CH_2_C*H*), 1.41 (br, 11H, 9H in C(C*H*_3_)_3_, 2H in CHC*H*_2_). Conversion 86%. DP (UV-vis) 45.

### Poly(acryloyl hydrazide) **P_*x*_**

In a typical experiment, trifluoroacetic acid (TFA) (15 mL) was added dropwise to poly(*tert*-butyl-2-acryloylhydrazine-1-carboxylate) (**Boc-P_40_**) (1.5 g) and the yellow solution was stirred at r.t. overnight. Excess of TFA was removed by blowing a steady stream of Argon and the resulting oil was diluted in water (15 mL). The **P_40_**·TFA salt formed was neutralised by adding NaHCO_3_ until no foaming was observed. The colourless solution was allowed to stir overnight. The crude polymer was purified by dialysis against water. The water was removed by lyophilisation and by drying in a desiccator with P_2_O_5_ to afford 650 mg of **P_40_** as a white powder (92%). ^1^H-NMR (300 MHz, D_2_O) *δ* (ppm) 1.59–2.08 (br m, (3·DP)H), 1.01 (s, 3H), 0.95 (s, 3H). ^13^C-NMR (100 MHz, D_2_O) *δ* (ppm) 174.9 (s), 40.2–40.5 (d), 34.4–35.7 (d). DP (^1^H-NMR) 40. IR (neat) *ν*_max_ 3254 (w br, N–H), 1609 (m br, C=O), 1428 (s sh) cm^–1^.

### Conjugation of poly(acryloyl hydrazide) **P_*x*_** with aldehydes

In a typical experiment, 200 μl of a 100 mM‡
‡100 mM in hydrazide moieties. Final concentration of hydrazides in solution = 50 mM. solution of **P_*x*_** in acetic acid (AcOH)/D_2_O buffer§
§AcOH/D_2_O buffer = 100 mM AcOH in D_2_O at pH 2.9. Other buffers used include 5% AcOH in D_2_O pH 2.9 (Table 3), 100 mM Na_2_HPO_4_ in D_2_O pH 9.1 (Table 3) and 95% DMSO-*d*_6_ 5% AcOH in D_2_O (Table 4, Tables S2–S4†) final pH = 2.9. was mixed with 200 μl of a 100 mM solution of the aldehyde in the required solvent.‡ This mixture was shaken at 60 °C for 24 h.¶
¶In aqueous conditions, samples can be incubated for only 2 h at r.t. Polymers were used without further purification.

## Results and discussion

### Poly(acryloyl hydrazide) synthesis

**Scheme 1:**
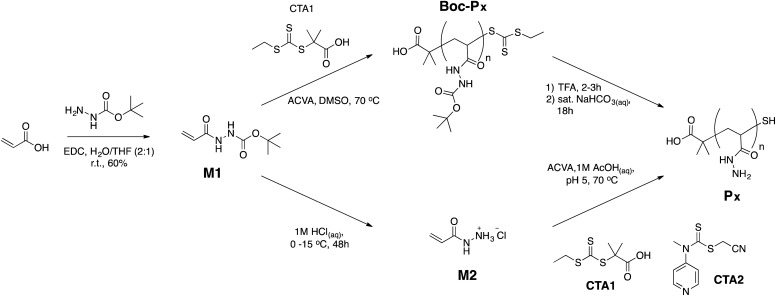
Synthetic strategies investigated for the synthesis of poly(acryloyl hydrazide).

Although several conditions have been reported in the literature for the synthesis of poly(acryloyl hydrazide) and poly(methacryloyl hydrazide),^29^,^32^–^34^ including the recently reported polymerisation of unprotected poly(methacryloyl hydrazide) at pH 0,^43^ we decided to explore a synthetic route that could avoid the use of an excess of toxic hydrazine. Two synthetic strategies to achieve the target poly(acryloyl hydrazide) **P_*x*_** starting from commercially available *tert*-butyl 2-acryloylhydrazine-1-carboxylate were thus investigated (Scheme 1). In both cases, reversible addition–fragmentation chain transfer (RAFT) polymerisation was employed because of its versatility for the preparation of acrylamide based polymers.^44^–^46^ Our initial attempts focused on the polymerisation of the deprotected acryloyl hydrazide (**M2**, Scheme 1, bottom). However, this strategy proved challenging. Initial polymerisation of this monomer was done with **CTA1**, a chain transfer agent previously used in our group,^47^ using acetate buffer (pH 5) at 70 °C. This pH was selected as a compromise to maximise solubility of the RAFT agent and protonation of the hydrazide. Although monomer consumption was observed rapidly by ^1^H NMR, no characteristic broad polymer peaks were seen in the expected alkyl region 1.9–1.1 ppm (Fig. S3, ESI†). Instead, the appearance of broad peaks at 2.86 and 2.21 ppm suggested the formation of oligomeric compounds, probably resulting from the Michael addition of the nitrogen in one hydrazide to the α,β-unsaturated system in another monomer. Moreover, the characteristic yellow colour of the solution due to **CTA1** also disappeared over time, suggesting that degradation of the transfer agent was occurring. We think both results indicate that possibly at this pH the hydrazide monomer was not fully protonated and thus remained strongly nucleophilic. Thus, we attempted the polymerisation using **CTA2**, a RAFT agent whose reactivity can be tuned as a function of the pH of the polymerisation solution.^48^ This RAFT agent is suitable for the polymerisation of challenging monomers and it could be used in aqueous conditions at low pH to ensure full protonation of the hydrazide monomer. Two conditions were thus explored ([monomer] : [**CTA2**] : [*p*-toluene sulfonic acid] at 100 : 1 : 1 and 100 : 1 : 200 ratios), but in both cases no polymer formation was observed.

**Fig. 1:**
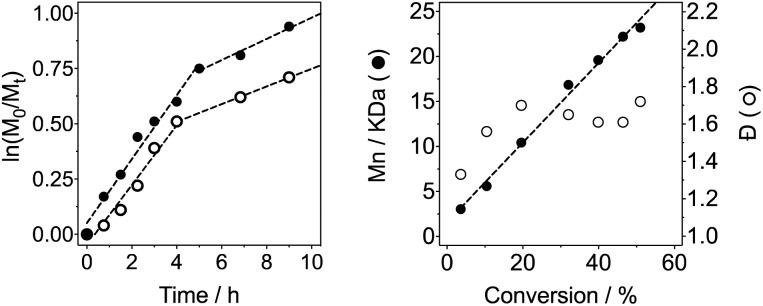
Left: Representative linear plot of ln[M]_0_/[M]_*t*_*vs.* time. Conditions: [black circle] [M] = 0.9 M, [M]/[CTA]/[V-501] = 100/1/0.2; ○: [M] = 0.9 M, [M]/[CTA]/[V-501] = 100/1/0.11. Right: Representative plot of measured number average molecular weight (*M*_n_) *vs.* conversion ([black circle]) and *Đ vs.* conversion (○). *M*_*t*_, *M*_n_ and *Đ* calculated by GPC using 0.005 M NH_4_BF_4_ in DMF at 50 °C as the eluent.

In light of these issues with the polymerisation of acryloyl hydrazide, the polymerisation of the protected monomer (**M1**) in DMSO at 70 °C was attempted instead. In this case, a decrease in alkene signals in ^1^H-NMR could be observed, as well as broadening of N–H signals and the *tert*-butyl signals, and the appearance of new broad signals in the alkyl region (Fig. S4, ESI†). To determine optimum reaction times, we carried out kinetic studies of the reaction by taking aliquots at different intervals and monitoring the conversion (*c*) by ^1^H-NMR. As expected for any free radical polymerisation, the reaction followed first order kinetics, at least during the initial stages of the polymerisation (Fig. 1). However, a deviation from linearity could be observed when the natural logarithm of the relative monomer concentration was plotted against time (Fig. 1A), suggesting termination may be occurring at later stages of the reaction. A similar behaviour has been observed in the polymerisation of other (meth)acrylamides,^49^ which has been assigned to the degradation of the polymer's trithiocarbamate end-group *via* an amide backbiting mechanism.^50^ In our case, a deviation from linearity was observed, even when polymerisations were performed at 50 °C and 44 °C, using suitable initiators for those temperatures (Fig. S7†). When the reaction was carried out at 30 °C, only 40% conversion was achieved after 24 h. Similarly, reduction of the amount of initiator used had no major effect on the kinetics of the reaction, beyond the appearance of a small induction period (Fig. 1A, ○). As in most previous cases, the reaction slowed down at higher conversions, suggesting termination. This effect agreed with the observed increase in the dispersity (*Đ*) in molecular weight as the polymerisation progressed (Fig. 1B).

**Fig. 2:**
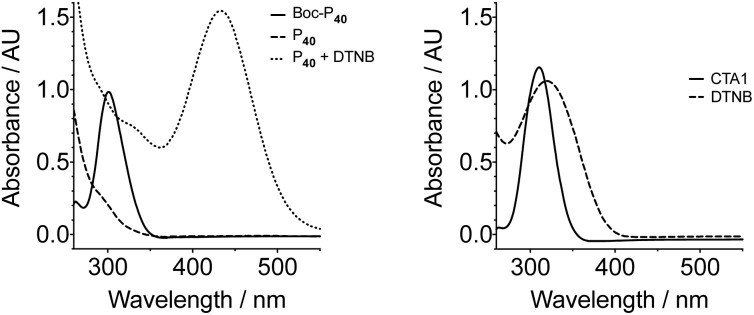
Representative UV-Vis spectra of the polymers prepared in this work and the reaction of the poly(acryloyl hydrazide) and DTNB. UV-Vis spectra for **CTA1** and DTNB are shown for comparison. Conditions: **Boc-P_40_** and **CTA1** were carried out in DMSO. **P_40_**, **P_40_** + DNTB and DTNB were carried out in water at r.t. [**Boc-P_40_**] = 0.63 mg ml^–1^, [CTA] = 0.03125 mg ml^–1^, [**P_40_**] = 1.33 mg ml^–1^, [DTNB] = 0.02 mg ml^–1^.

Nonetheless, the tested conditions allowed us to predict the molecular weight of the formed polymers (Fig. 1B), and thus we synthesised different polymer batches with degrees of polymerisation (DPs) ranging from 43 to 127 and dispersities between 1.38–1.51 (Table 1). Attempts to prepare polymers of larger DPs (*i.e.* ∼200 monomer units) lead to polymers with higher *Đ* values, despite having reached similar monomer conversions than the other polymerisations performed (Table 1). UV-Vis analysis of all polymers revealed the presence of a characteristic band at around 300 nm (Fig. S5, ESI†), in close proximity to that of the transfer agent used (Fig. 2). The presence of this band suggested that all polymers still retained some of the RAFT agent used. There were small differences in the wavelength for maximum absorption (*λ*_max_) implying that the chemical environment around this chain transfer agent was changing as the degree of polymerisation was increased.

**Table 1:** Boc-protected poly(acryloyl hydrazide)s (**Boc-P_*x*_**) described in this paper

Polymer	[M]/[CTA]	*c* ^*a*^	DP_th_ ^*b*^	*M*_n_ ^*c*^	*Đ* ^*c*^
**Boc-P_40_**	50	86%	43	9810	1.38
**Boc-P_80_**	99	79%	78	20 306	1.52
**Boc-P_130_**	151	84%	127	31 552	1.51
**Boc-P_170_**	195	87%	170	44 826	1.95

^*a*^Conversion (*c*) calculated from ^1^H NMR peak integration of alkene signals *versus* a known standard.

^*b*^[M]/[CTA] × *c*.

^*c*^Calculated by GPC using 0.05 M LiBr in DMF at 60 °C as the eluent.

The polymers were then deprotected by reacting in neat TFA for 2–3 h followed by dilution in water and saturation with NaHCO_3_. This way the excess of TFA was neutralised and the hydrazides deprotonated to afford the target poly(acryloyl hydrazide) **P_*x*_**. We also observed the loss of the thiocarbonylthio group to afford a thiol, possibly because of the strongly basic conditions employed during neutralisation (pH NaHCO_3_(sat) ∼12). For example, the UV-Vis signal at 300 nm observed for **Boc-P_40_** (Fig. 2, solid line), could not be observed for the deprotected polymer **P_40_** (Fig. 2, dashed line). Moreover, when this deprotected polymer was reacted with 5,5′-dithiobis(2-nitrobenzoic acid) (DNTB), a colorimetric dye used for the identification of thiols,^51^ a characteristic peak at 435 nm could be observed (Fig. 2, dotted line), representative of the formed 5-mercapto-2-nitrobenzoic acid. No effect over the molecular weight distribution of this polymer could be observed following basic treatment, suggesting that coupling of the polymer chains through disulfide oxidation was minimal under these conditions (Fig. S8†).

NMR analysis of the target poly(acryloyl hydrazide)s **P_*x*_** confirmed the absence of the Boc signal (Fig. S6, ESI†). Moreover, signals corresponding to the methyls originating from the RAFT agent could now be clearly identified at 0.94 and 1.00 ppm, and were used to determine the degree of polymerisation (Table 2). Again, these values were in close agreement to those expected from the conversion during the polymer synthesis, validating the use of the described conditions for the synthesis of these polymers.

**Table 2:** Poly(acryloyl hydrazide)s (**P_*x*_**) described in this paper

Polymer	DP_NMR_ ^*a*^	*M*_n_ ^*b*^	*Đ* ^*b*^
**P_40_**	49	10 918	1.37
**P_80_**	106	18 446	1.33
**P_130_**	136	—^*c*^	—^*c*^
**P_170_**	162	—^*c*^	—^*c*^

^*a*^Calculated from ^1^H NMR peak integration of methyl signals at 0.94 and 1.00 ppm *versus* alkyl backbone.

^*b*^Calculated by GPC.

^*c*^Samples were not soluble in GPC eluent.

### Aldehydes coupling to polymer

**Fig. 3:**
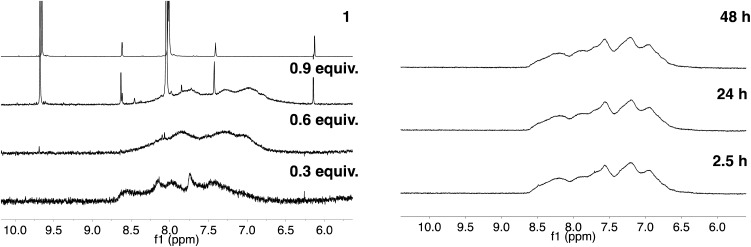
Left: ^1^H NMR spectra of **1** (Top) and of **P_40_** treated with different amounts of **1** after 1 h of reaction. Adapted with permission from ref. 27. Right: ^1^H NMR spectra of the reaction of **P_40_** with 0.6 eq. **1** analysed at different intervals.

As described in our previous work with siRNA, 4-imidazolecarboxaldehyde (**1**) was used as a model hydrophilic aldehyde to identify conditions for the functionalisation of the polymer scaffold.^27^ As reported, the coupling of **1** with poly(acryloyl hydrazide) **P_*x*_** could be performed in 100 mM AcOH/D_2_O buffer with varying amounts of aldehyde in relative short times (1–4 h). When we looked at these conditions in more detail, we identified that the duration of the coupling was not dependent on the number of equivalents added, with 0.3 and 0.6 eq. of **1** requiring 1 h for a complete coupling to be observed (Fig. 3, Table S1†).^27^ For 0.9 eq., no full consumption of aldehyde was observed even after 24 h (Fig. S9, ESI†). However, for these equivalents, the amount of free aldehyde (∼27%) was more or less constant at different intervals, suggesting that ∼65–66% of the hydrazide side-chains had reacted. This degree of functionalisation is what we had reported before and in agreement with other examples in the literature.^27^,^29^

As just discussed, the degree of functionalisation remained constant under the conditions (100 mM AcOH in D_2_O at pH 2.9) used for the functionalisation, and no regeneration of the aldehyde was observed. To further probe this, **P_40_** was incubated with 0.6 eq. of 4-imidazolecarboxaldehyde (**1**), enough aldehyde to ensure full reactivity with the polymer backbone. The degree of functionalisation was evaluated by NMR over a period of 48 h, and no signal for the free aldehyde was observed at any point of the experiment (Fig. 3, right). A similar effect was observed when the degree of functionalisation was monitored for the reaction with 1 eq. of the aldehyde, with no significant changes in the amount of free aldehyde observed with time (Fig. S10†). These experiments suggested that the system had reached thermodynamic equilibrium, and any aldehyde dissociation would be compensated by the reformation of hydrazone. Remarkably, when the samples were diluted twofold following initial incubation for 2 h, no regeneration of the aldehydes could be observed, suggesting that the thermodynamic equilibrium was not significantly affected under these conditions (*i.e.* 100 mM AcOH in D_2_O at pH 2.9 and twofold dilution) (Fig. S11†).

We decided then to investigate the coupling of a series of hydrophilic aldehydes including anionic glyoxylic acid (**2**) neutral glyceraldehyde (**3**), or biologically similar betaine aldehyde chloride (**4**), pyridoxal-5′-phosphate (**5**) and 5-formyluracil (**6**) (Table 3). As expected, the coupling was highly dependent on the solubility of the aldehydes and/or the polymers obtained in the buffer used. When acidic conditions were employed (in this case 5% AcOH, 24 h incubation. See discussion on organic solvent for details), only the neutral and cationic aldehydes **3** and **4** could give similar degrees of functionalisation to that reported for the imidazole derivative (**1**). Anionic aldehyde **5** and the uracil derivate **6** were insoluble in the acidic buffer employed while glyoxylic acid (**2**) resulted in insoluble polymers that compromised the characterisation of the degree of functionalisation. Switching to a basic buffer (100 mM Na_2_HPO_4_ in D_2_O pH 9.1) compromised the overall coupling, and in this case, only anionic derivatives gave satisfactory degrees of functionalisation ranging from 63% for **2** to a very good 86% in the case of the phosphate derivative **5**. Interestingly, uracil derivative **6** remained insoluble in any of the conditions tested and we decided to explore the use of a polar organic solvent like DMSO to carry out the reactions. However, the prepared poly(acryloyl hydrazide)s **P_*x*_** showed very low solubility in this solvent and it had to be dissolved in aqueous conditions before any further dissolution with DMSO. Yet this way we could prepare solutions of **P_40_** with up to 95% of DMSO, without any obvious formation of precipitates by visual inspection.

**Table 3:** Percentage loading in coupling reactions of **P_40_** with 1 eq. of water soluble aldehydes under different aqueous conditions

Entry	Aldehyde	5% AcOH in D_2_O pH 2.9	100 mM Na_2_HPO_4_ in D_2_O pH 9.1	95% DMSO-*d*_6_ 5% AcOH in D_2_O
**P_40_1**	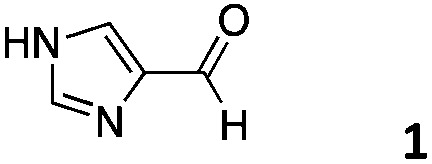	66%	—	74%
**P_40_2**	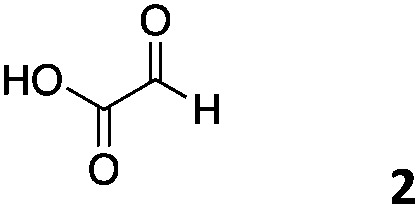	3%	63%	68%
**P_40_3**	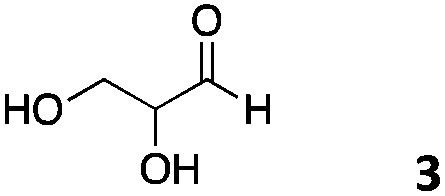	65%	13%	20%
**P_40_4**	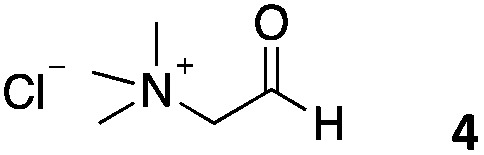	80%	—	30%
**P_40_5**	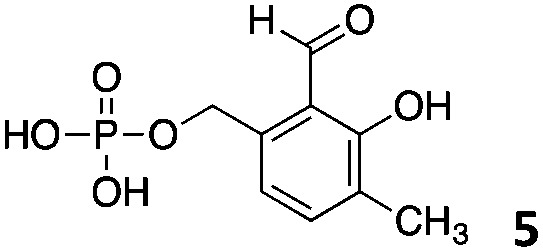	—^*a*^	86%	—^*a*^
**P_40_6**	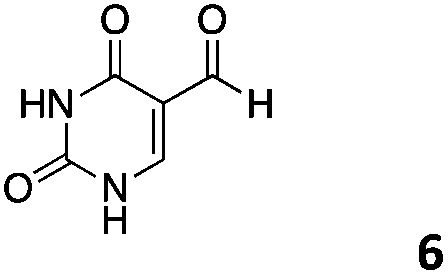	—^*a*^	—^*a*^	65%

^*a*^Insoluble aldehyde and/or insoluble products.

The use of an organic co-solvent like DMSO opened new possibilities but required exploring the effect that this solvent had in the coupling conditions. Investigation of the kinetics of the reaction suggested that now the coupling of the hydrazides and the aldehydes was much slower and often long incubation at 60 °C was required to achieve similar degrees of functionalisation than those observed under aqueous conditions. For instance, when **P_40_** and 1 eq. of our model aldehyde **1** were dissolved in a 1 : 1 mixture of aqueous buffer (5% AcOH in D_2_O) and DMSO-*d*_6_, less than half of the aldehyde had coupled after incubation for 24 h at 60 °C (Fig. S12, ESI†). This could be improved by increasing the amount of DMSO-*d*_6_ to 95%, with approximately 76% of the aldehyde reacting in this case (Fig. S13, ESI†). In view of this effect of the organic solvent in the rate of functionalisation, all the couplings reported in Table 3 were performed following 24 h incubation at 60 °C.

Using these conditions (95% DMSO-*d*_6_/5% AcOH in D_2_O) we could functionalise **P_40_** with the uracil derivative **6**, reaching similar levels of functionalisation (65%) to the previous cases (Table 3). These conditions proved to be quite versatile and all aldehydes except for the phosphate derivative **5** could be dissolved in this solvent. Yields varied again, with glyceraldehyde **3** giving a surprising low degree of functionalisation (20%). We believe that in this case functionalisation of the polymer is outcompeted by the self-polymerisation of **3***via* cyclic ketal formation, in agreement with the disappearance of the aldehyde signals and the appearance of a new signal at 3.53 ppm (Fig. S14, ESI†).

The use of an organic solvent opened also the possibility of evaluating the coupling conditions for hydrophobic aldehydes, often present in biologically relevant polymers such as gene vectors or antimicrobial polymers. A series of commercially available aldehydes ranging from aliphatic to aromatic aldehydes (Table 4) were evaluated for coupling to poly(acryloyl hydrazide) **P_40_** using 95% DMSO-*d*_6_/5% AcOH in D_2_O as the reacting buffer. Overall, coupling efficiency for the aromatic aldehydes was around 70% regardless of the size of the aromatic aldehyde used (*e.g.***P_40_7***vs.***P_40_8**). We could efficiently incorporate substituted aromatics, including hydroxylated (**P_40_9–P_40_11**), carboxylated (**P_40_12**) and fluorinated aldehydes (**P_40_14** and **P_40_15**). The degree of substitution seemed to have an effect in the coupling of the hydroxylated aromatics, with only 50% loading observed for 2,4,5-trihydroxybenzaldehyde (**11**). A similar effect was observed for the fluorinated derivatives, suggesting both electronic and steric effects should be contributing to the efficiency of the functionalisation. Steric effects were more evident for the aliphatic aldehydes, with acetaldehyde (**16**) reaching almost a 90% of loading. Isovaleraldehyde (**17**) gave a smaller degree of functionalisation (82%) with all of the other aliphatic aldehydes showing significantly less loading. However, solubility of the formed polymers with these long aliphatic aldehydes (**18–20**) was low, compromising the characterisation by NMR to determine the percentage of loading for these aldehydes. Nevertheless, broadening of ^1^H-NMR peaks belonging to the aliphatic chains supported evidence for hydrazone formation (Fig. S15, ESI†). Some of these experiments were then repeated under the same conditions but using twice the amount of aldehyde (*i.e.* 2 eq.). Overall, increasing the amount of aldehyde had a beneficial effect over the coupling efficiency, which in some cases reached almost full conversion (Table S2, ESI†).

**Table 4:** Percentage loading in coupling reactions of **P_40_** with 1 eq. of different hydrophobic aldehydes

Entry	Aldehyde	Loading	Entry	Aldehyde	Loading
**P_40_7**	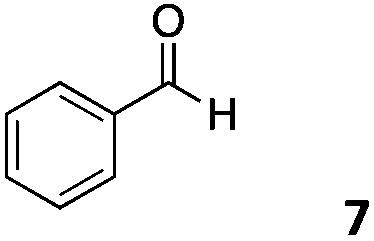	64%	**P_40_14**	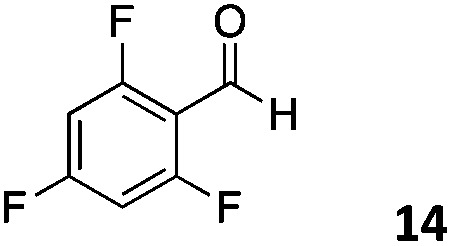	72%
**P_40_8**	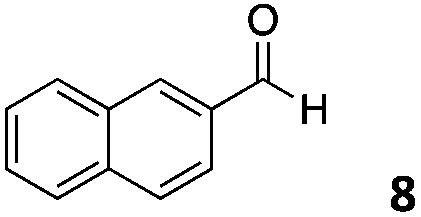	73%	**P_40_15**	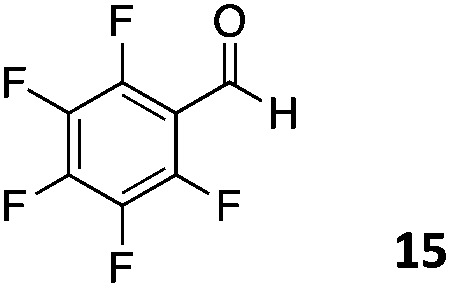	56%
**P_40_9**	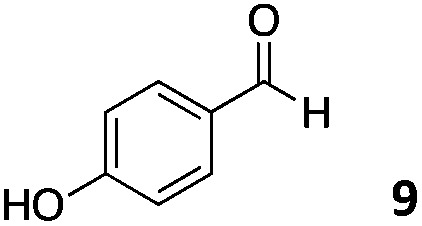	71%	**P_40_16**	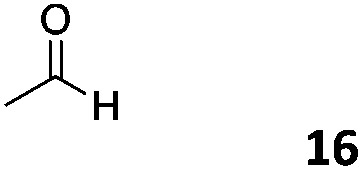	89%
**P_40_10**	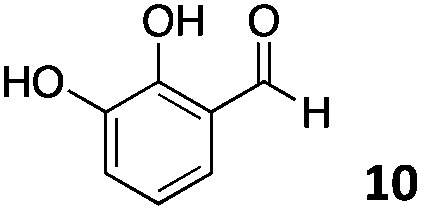	85%	**P_40_17**	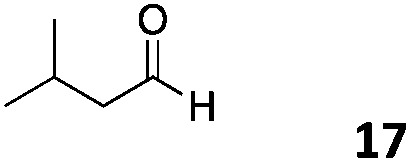	82%
**P_40_11**	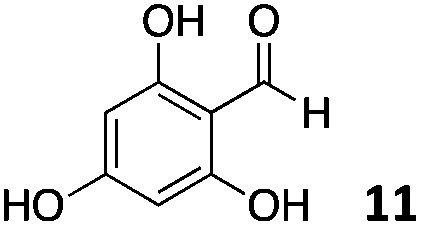	50%	**P_40_18**	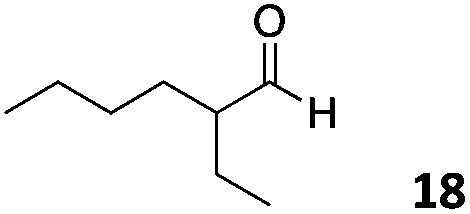	—^*a*^
**P_40_12**	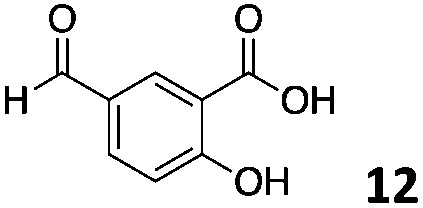	75%	**P_40_19**	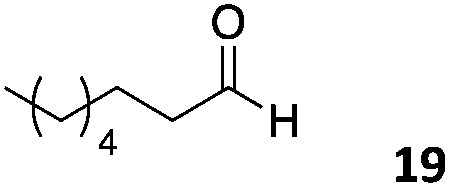	62%^*a*^
**P_40_13**	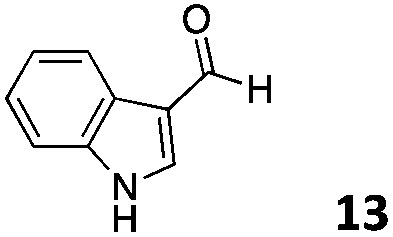	52%	**P_40_20**	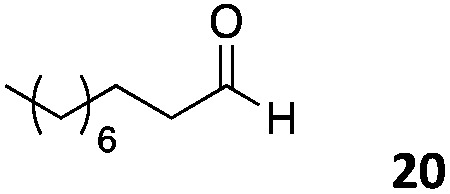	59%^*a*^

^*a*^Insoluble products.

### Impurities formed after coupling

During the coupling reaction with aromatic aldehydes a new sharp signal was identified in the NMR spectra (Fig. S15, ESI†). These sharp singlets between 9.0–8.5 ppm are consistent with *H*-C(R) = X environments, and would suggest the formation of imines or hydrazones, through cleavage of the NH–NH bond or the CO–NH bond respectively (Scheme S1†). We therefore carried out coupling reactions with representative aldehydes and hydrazine monohydrate or ammonia respectively, using 95% DMSO-*d*_6_/5% AcOH in D_2_O as the reacting buffer. Coupling between hydrazine and benzaldehyde, and between hydrazine and 4-hydroxybenzaldehyde, resulted in the formation of hydrazones with NMR spectra that matched those of the impurities observed (Fig. S16, ESI†). These two impurities could be identified in all the couplings with aromatic aldehydes, although in most cases the impurity's content was generally around 1–3% of total amount of aldehyde used (Table S3, ESI†). When coupling experiments with sub-stoichiometric (0.25 eq.) amounts of benzaldehyde were performed, both mono- and di-hydrazone were observed by NMR, even before all the aldehyde had coupled (∼4 h) (Fig. S17, ESI†). The initial amount of impurity was very small (<2% of the initial aldehyde). Once all the aldehyde had been consumed we observed a steady increase in the concentration of mono-hydrazone (*e.g.* benzylidenehydrazine), and up to 13% of the initial aldehyde had converted into this compound after 12 days. No significant increase in the intensity of the di-hydrazone (*e.g.* 1,2-dibenzylidenehydrazine) was observed and remained around 3% even after 12 days.

At this stage, the mechanism of this side-reaction is unclear, but we believe that the mono-hydrazone is formed through cleavage of the C–N bond in the polymer's hydrazone side-chains (Scheme S1†). The di-hydrazone will be then formed by subsequent reaction between any free aldehyde and the mono-hydrazone until no free aldehyde is present. We anticipate that cleavage will be probably facilitated by the presence of nucleophilic hydrazides in the vicinity of the hydrazone, and thus the amount of impurity is only significant when sub-stoichiometric amounts of aldehydes are employed.

### Effect of *M*_w_ on coupling

Finally, to explore the effect of polymer size on this post-polymerisation strategy, functionalisation of **P_80_** using 95% DMSO-*d*_6_/5% AcOH in D_2_O as the reacting buffer was performed (Table S4, ESI†). Overall coupling efficiencies were very similar to those reported for **P_40_**, although bulky and aliphatic aldehydes yielded lower degrees of functionalization. This was particularly the case for 2-naphthaldehyde (**8**) and acetaldehyde (**16**) with over 20% lower coupling efficiencies than that observed for **P_40_** (Table 4). Similarly to **P_40_**, coupling efficiency with **P_80_** could be improved by reacting with 2 eq. of aldehyde with, on average, an additional 10% of the side-chains functionalized this way. Kinetic studies were carried with several aldehydes and **P_80_** to investigate the time required to reach maximum possible conversion for each substrate. Most of the aldehydes reacted within 6 h, with acidic 5-formylsalicylic (**12**) and aliphatic isovaleraldehyde (**17**) reaching their highest loading efficiency in less than 2 h. On the other hand, indole-3-carboxaldehyde (**13**) required at least 24 h reaction time to reach maximum conversion. The level of impurities formed in theses couplings to **P_80_** (Table S4, ESI†) were overall similar to those reported for **P_40_**, being around 1–3% of the total amount of aldehyde used in most cases.

## Conclusions

Here, we have presented the synthesis and post-polymerisation functionalisation of poly(acryloyl hydrazide). RAFT polymerisation was employed to prepare a small library of Boc-protected poly(acryloyl hydrazide)s. Following deprotection under acidic conditions, we demonstrated that poly(acryloyl hydrazide) is a versatile reactive scaffold that can mediate the synthesis of polymers carrying a wide range of functionalities, including non-water soluble and biologically similar aldehydes. The efficiency of the hydrazide-aldehyde coupling was modulated by tuning the reaction conditions, including the use of both aqueous and organic conditions, to yield polymers with a consistent degree of functionalisation. We believe that our results will be of relevance to those screening for activity in polymers with high and/or complex patterns of functionalisation, such as those working in the discovery of polymeric materials for biomedical applications. Our efforts to elucidate the nature and mechanism of the side-reaction identified, the optimisation of the synthetic route, as well as further applications will be reported in due course.

## Contributions

All authors contributed to the experimental set-up and discussed the results. DNC and FFT designed the polymer synthesis and characterisation. BM carried out GPC analysis of kinetic studies, and DNC and OC performed all other GPC analysis. DNC, JM and FFT designed the functionalisation experiments. DNC, OC, RB and JLB performed kinetic and stability experiments. DNC performed all of the other experiments. JM and FFT secured funding. DNC and FFT analysed the data and wrote the paper, with all other authors contributing to the final version of the manuscript.

## Conflict of interest

There are no conflicts of interest to declare.

## Supplementary Material

Supplementary information
