# Supplementary material for: Poly(acryloyl hydrazide), a versatile scaffold for the preparation of functional polymers: synthesis and post-polymerisation modification
Source: Polym Chem. 2017 Jul 3;8(31):4576–84. doi: 10.1039/c7py00535k (PMC6091239; doi:10.1039/c7py00535k)
Supplement: Supplementary file 1 [file PY-008-C7PY00535K-s001.pdf]

Electronic Supplementary Material (ESI) for Polymer Chemistry.
This journal is © The Royal Society of Chemistry 2017

Poly(acryloyl hydrazide), a versatile scaffold for the preparation of functional polymers: Synthesis and post-polymerisation modification.

Daniel N. Crisan[a], Oliver Creese[a], Ranadeb Ball[a], Jose Luis Brioso[a], Ben Martyn[b], Javier Montenegro[c,*] and Francisco Fernandez-Trillo[a,*].

[a] School of Chemistry, University of Birmingham B15 2TT (UK) E-mail: f.fernandez-trillo@bham.ac.uk [b] School of Chemistry, University of Warwick CV47AL (UK).
[c] Departamento de Química Orgánica y Centro Singular de Investigación en Química Biolóxica e Materiais Moleculares (CIQUS), Universidade de Santiago de Compostela E-15782 (Spain). E-mail: javier.montenegro@usc.es

Supplementary Figures and Tables

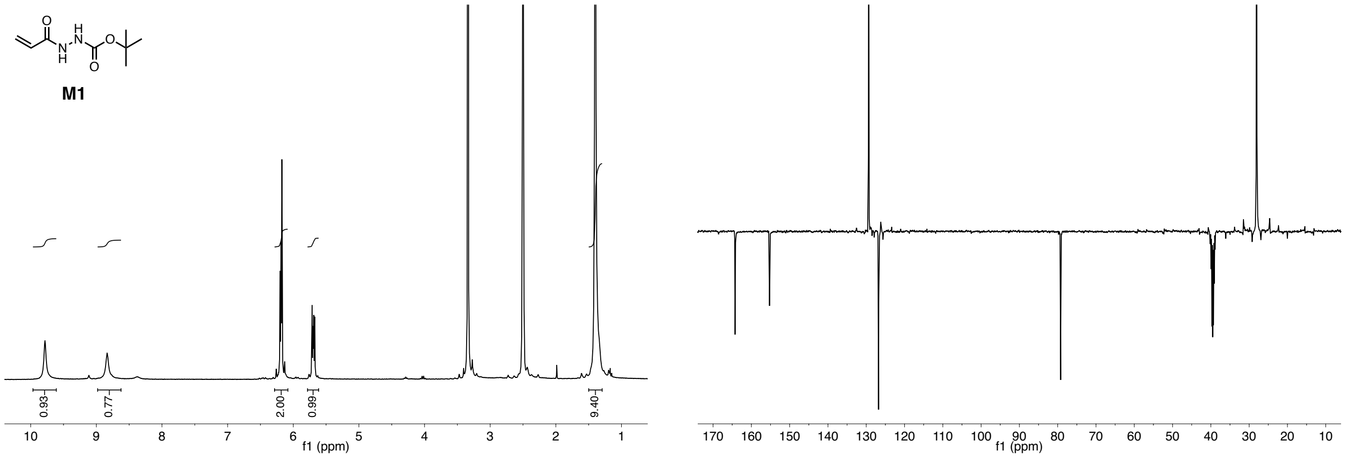

Fig. S1 ^{1}H-NMR and PENDANT[‡] ^{13}C-NMR spectra in DMSO-*d6* for *tert*-butyl 2-acryloylhydrazinecarboxylate (**M1**).

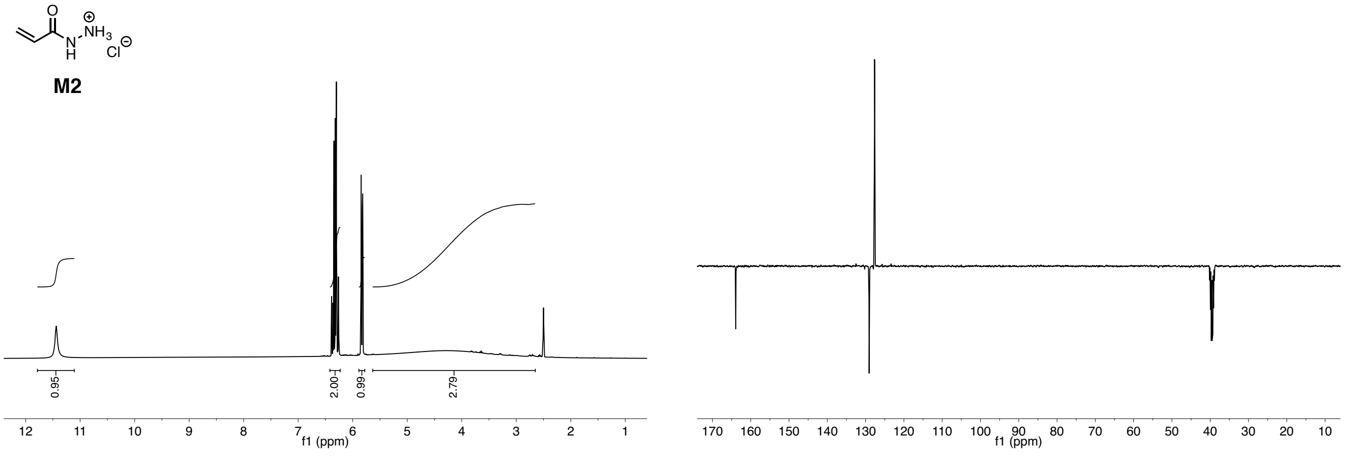

Fig. S2 ^{1}H-NMR and PENDANT ^{13}C-NMR spectra in DMSO-*d6* for acryloyl hydrazide (**M2**).

‡ PENDANT: Polarization enhancement nurtured during attached nucleus testing

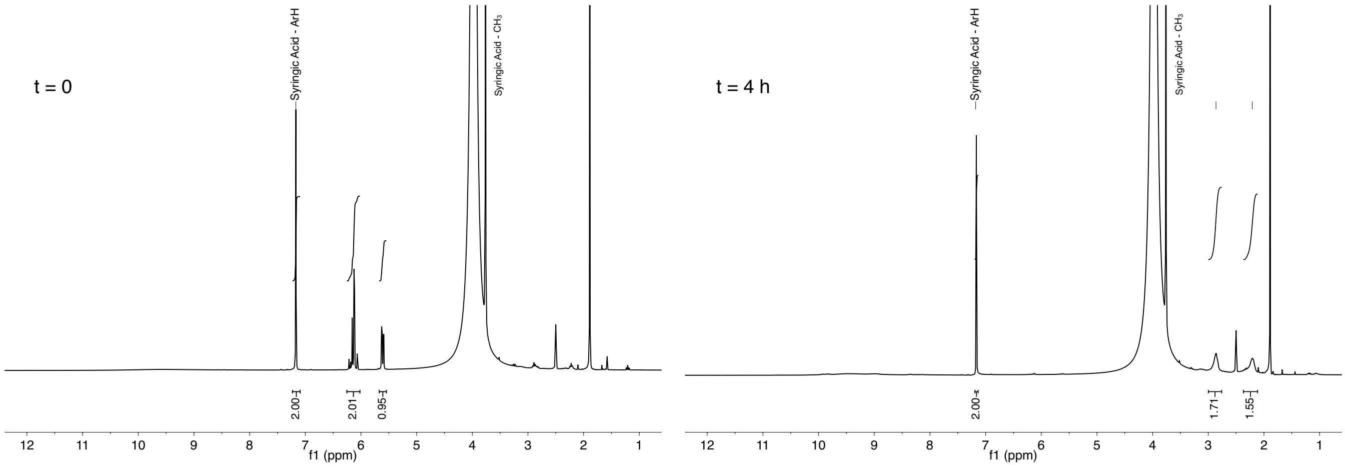

Fig. S3 ^{1}H-NMR spectra of a representative polymerisation of acryloyl hydrazide (**M2**) with **CTA1** in 1M acetate buffer pH 5 at 70 °C. The NMR samples were spiked with syringic acid as an internal standard. The integration of the monomer's alkene peaks (6.18 and 5.68 ppm) was compared to the integration of the aromatic peak of syringic acid (7.17 ppm) to obtain the % conversion.

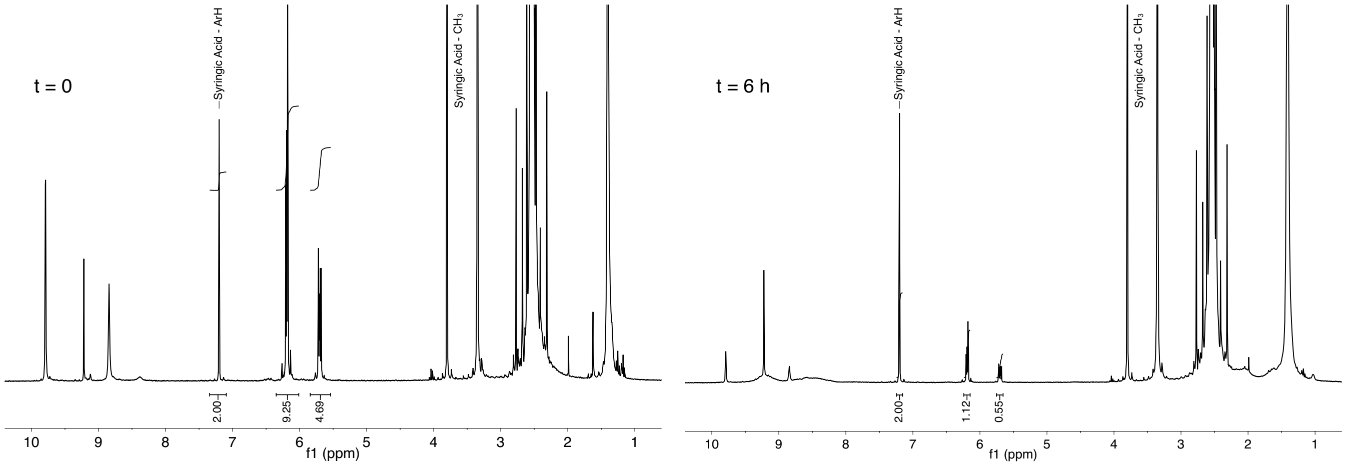

Fig. S4 ^{1}H-NMR spectra of a representative polymerisation of *tert*-butyl 2-acryloylhydrazinecarboxylate (**M1**) with **CTA1** in DMSO at 70 °C. The NMR samples were spiked with syringic acid as an internal standard. The integration of the monomer's alkene peaks (6.18 and 5.68 ppm) was compared against the integration of the aromatic peak of syringic acid (7.20 ppm) to obtain the % conversion.

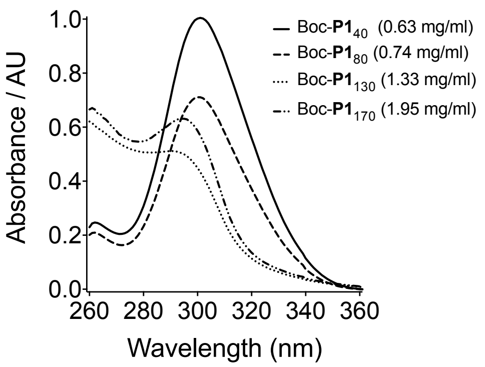

Fig. S5 UV-Vis spectra of the Boc-protected polymers prepared in this work. All samples measured in DMSO at r.t.

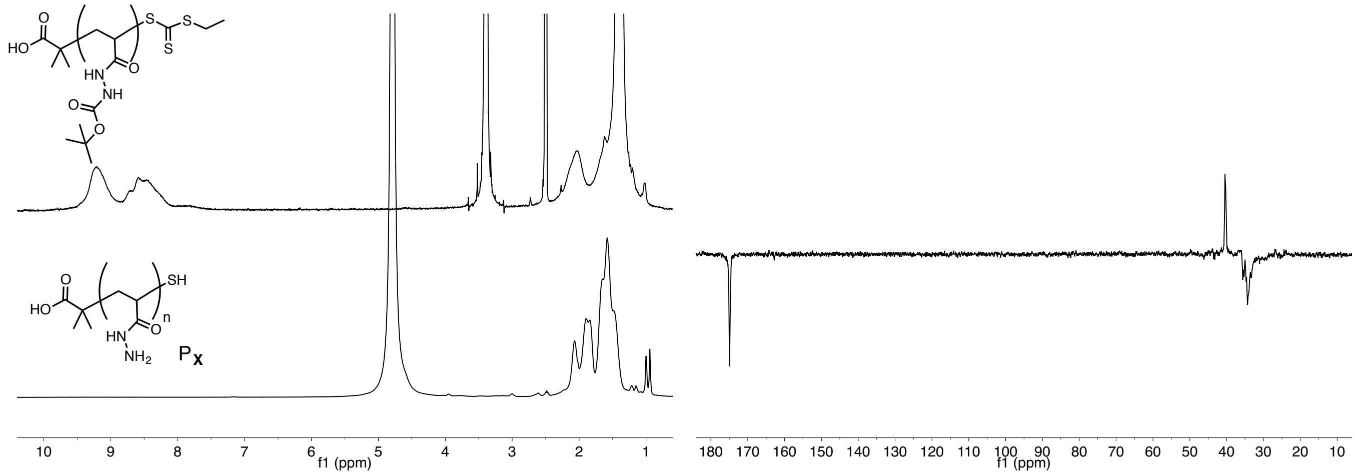

Fig. S6 Left: Representative ^{1}H-NMR spectra of Boc-$\mathbf{P_{40}}$ (top) in DMSO-*d6* and poly(acryloyl hydrazide) $\mathbf{P_{40}}$ after deprotection with TFA (bottom) in D_2O. Right: Representative PENDANT ^{13}C-NMR s spectra of poly(acryloyl hydrazide) $\mathbf{P_{40}}$ in D_2O.

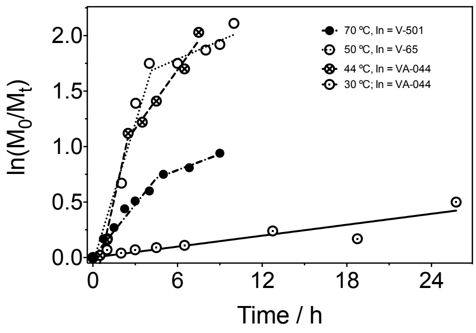

Fig. S7 Linear plot of $\ln[M]_0/[M]_t$ vs. time for polymerisations performed at different temperatures. Conditions: [M]=0.9M, [M]/[CTA]/[In]=100/1/0.2. 4,4'-Azobis(4-cyanovaleric acid) (**V-501**), 2,2'-Azobis(2,4-dimethylvaleronitrile) (**V-65**), and 2,2'-Azobis[2-(2-imidazolin-2-yl)propane]dihydrochloride (**VA-044**).

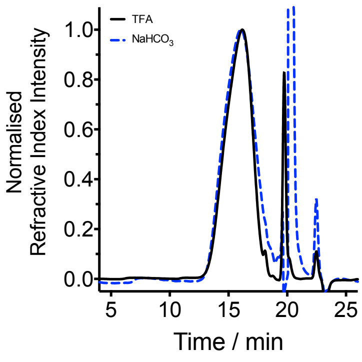

Fig. S8 GPC chromatograms of $\mathbf{P_{40}}$ before and after treatment with $NaHCO_3$. Conditions: 100 mM AcOH (aq) pH 2.9.

Table S1 Percentage of loading of $\mathbf{P_{40}}$ with 4-imidazolecarboxaldehyde (**1**) using different equivalents and different incubation times.

Equivalence of 4-imidazolecarboxaldehyde	Reaction Time (h)	% conjugation
**0.3**	1	30.0
	4	30.0
**0.6**	1	59.9
	4	60.0
	48	55.8
**0.9**	1	65.7
	4	65.7
	24	65.7

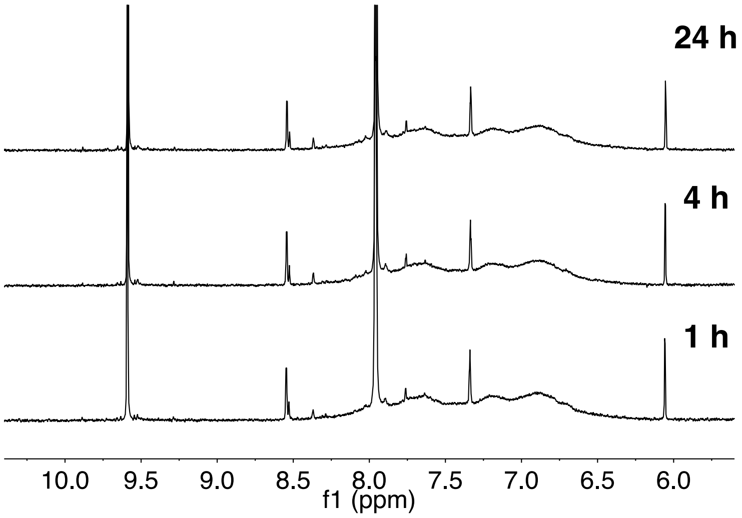

Fig. S9 Representative ^{1}H-NMR spectra of the reaction of $\mathbf{P_{40}}$ with 0.9 eq. of 4-imidazolecarboxaldehyde (**1**) at different reaction times.

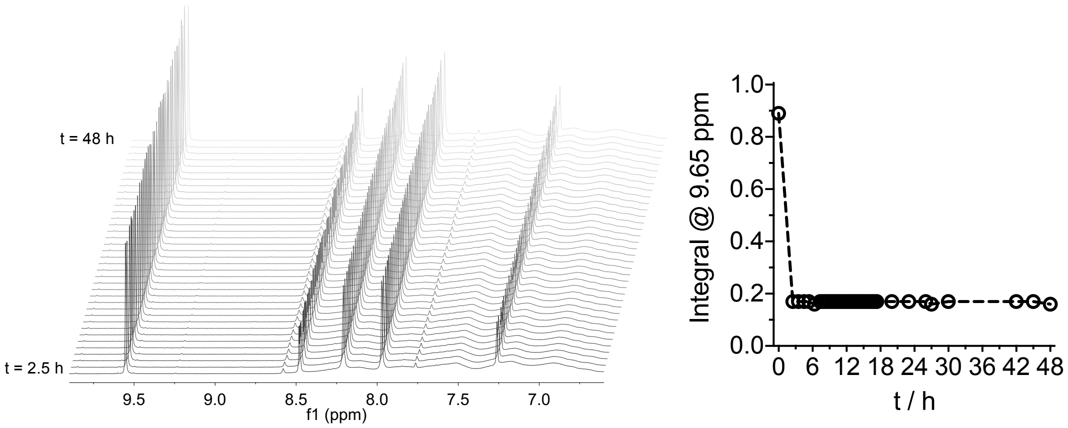

Fig. S10 Left: ^{1}H NMR spectra of the reaction of $\mathbf{P_{40}}$ with 1 eq. of 4-imidazolecarboxaldehyde (**1**) analysed at different intervals. Right: Change of integral value for the signal corresponding to the aldehyde (9.65 ppm) as a function of time. Sample were incubated for 2 h at r.t. prior to NMR analysis.

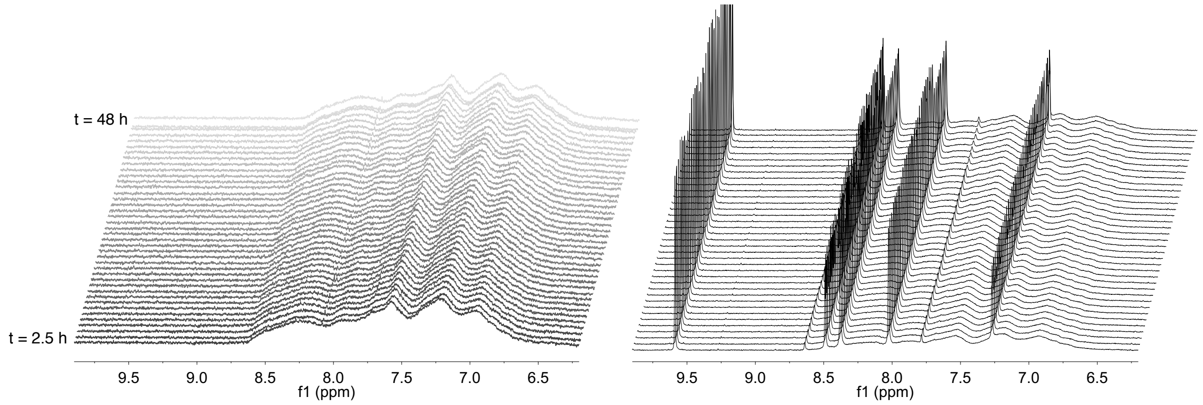

Fig. S11 Left: ^{1}H NMR spectra of the reaction of $\mathbf{P_{40}}$ with 0.6 eq. of 4-imidazolecarboxaldehyde (**1**) analysed at different intervals. Right: ^{1}H NMR spectra of the reaction of $\mathbf{P_{40}}$ with 1 eq. of 4-imidazolecarboxaldehyde (**1**) analysed at different intervals. In both cases, samples were incubated for 2 h at r.t. and diluted two fold prior to NMR analysis.

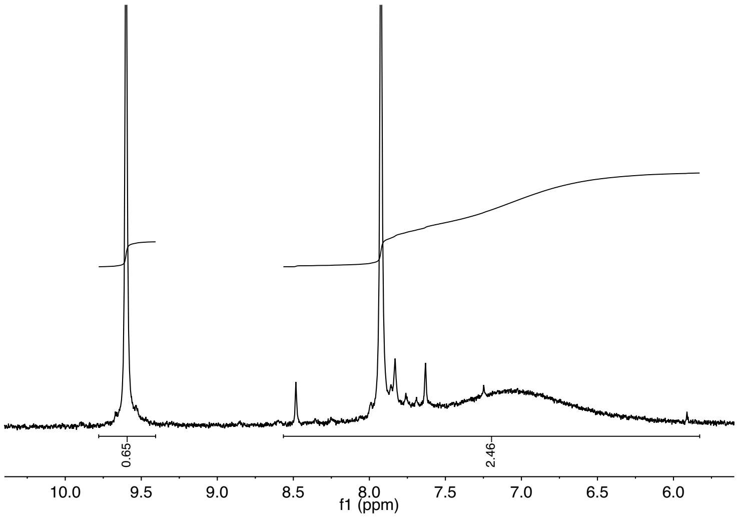

Fig. S12 Representative ^{1}H-NMR spectra of the functionalisation of $\mathbf{P_{40}}$ with 1.0 eq. of 4-imidazolecarboxaldehyde (**1**) in 50% aqueous buffer (5% AcOH in D_2O) 50% DMSO-*d*6 after 24 h incubation at 60 ^{o}C.

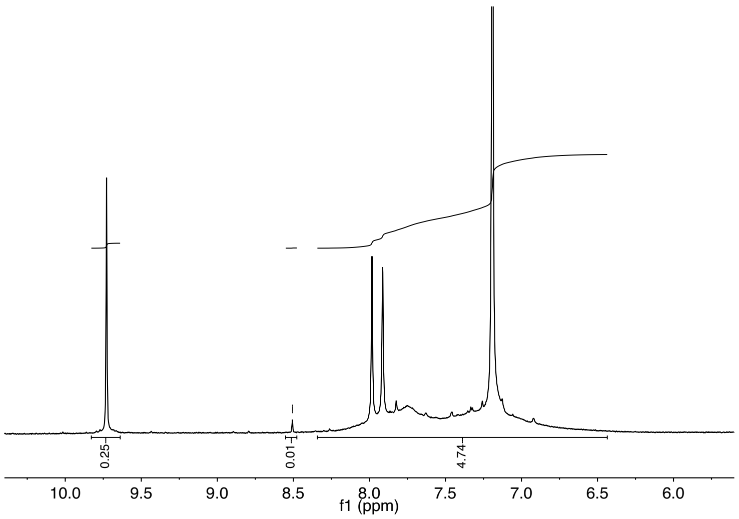

Fig. S13 Representative ^{1}H-NMR spectra of the functionalisation of $\mathbf{P_{40}}$ with 1 eq. of 4-imidazolecarboxaldehyde (**1**) in 95% DMSO-*d6* / 5% AcOH in D_2O. The sample was spiked with equimolar amounts of syringic acid. The integration values of the aromatic syringic acid signals (7.20 ppm) and imidazole signals for the free and conjugated aldehyde were compared to the free aldehyde CHO signal.

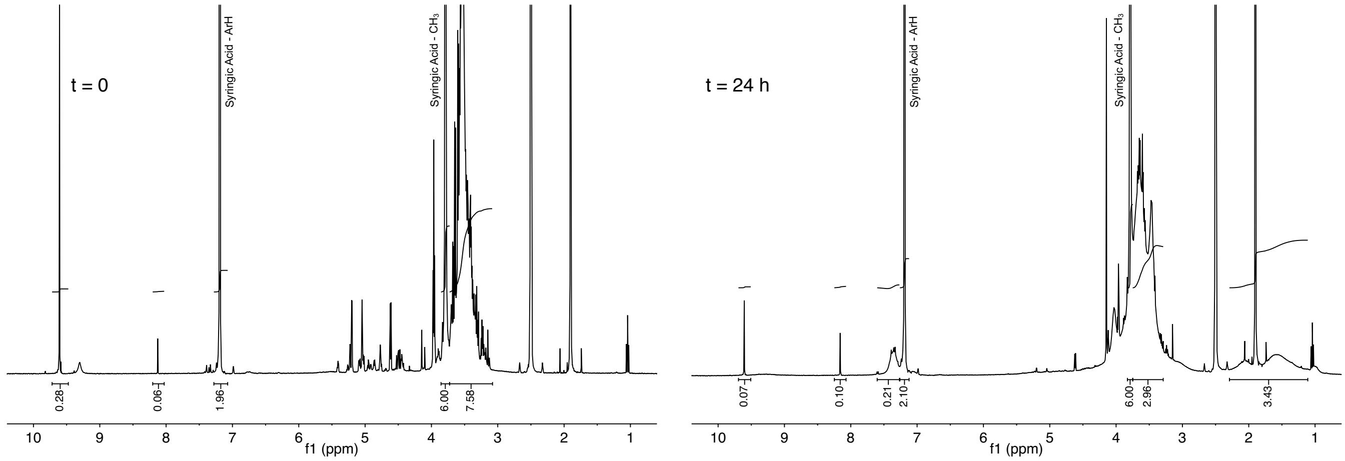

Fig. S14 Representative ^{1}H-NMR spectra of glyceraldehyde (left) and the reaction of $\mathbf{P_{40}}$ with 1.0 eq. of glyceraldehyde (**3**) in 95% DMSO-*d6* / 5% AcOH in D_2O (right). The samples were spiked with equimolar amounts of syringic acid. The integration values of the methoxy syringic acid signals (3.79 ppm) and glyceraldehyde signals for free and conjugated were compared to the free aldehyde signal and the hydrazone signal (7.38 ppm). The broad signal observed at 3.52 ppm for glyceraldehyde (**3**) corresponds to the self-condensation of this aldehyde under these conditions, which is reduced as the functionalisation progresses.

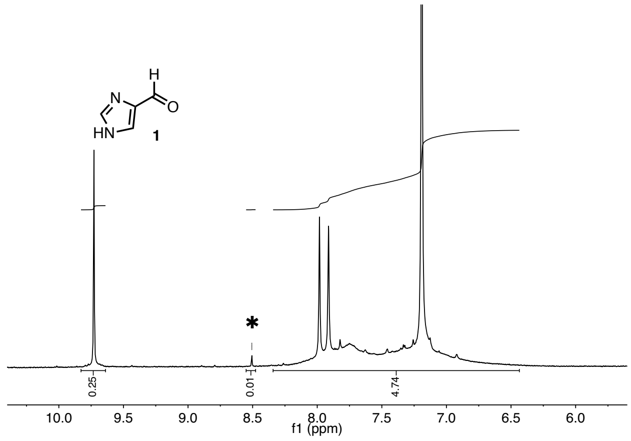
1
0.25
0.01
4.74
f1 (ppm)

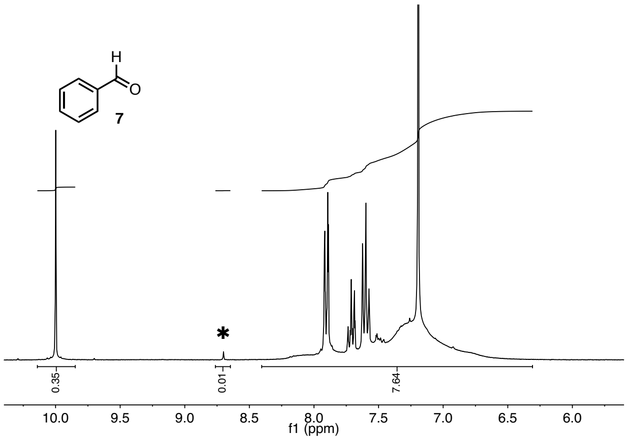
7
0.35
0.01
7.64
f1 (ppm)

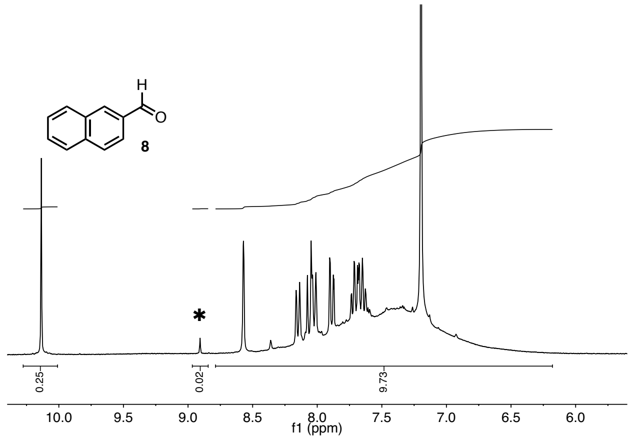
8
0.25
0.02
9.73
f1 (ppm)

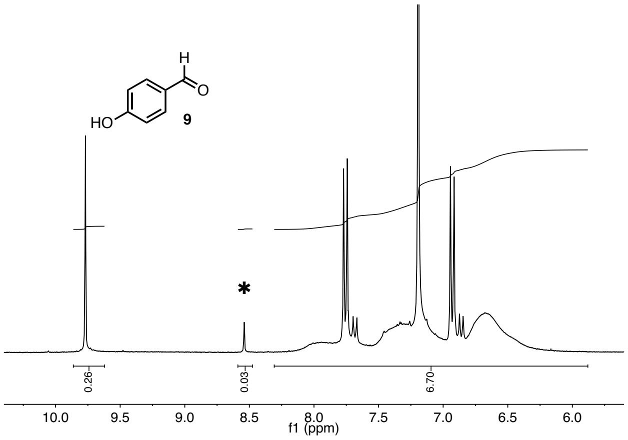
9
0.26
0.03
6.70
f1 (ppm)

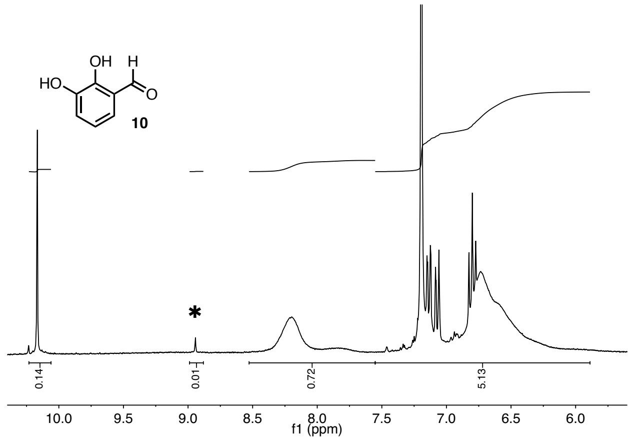
10
0.14
0.01
0.72
5.13
f1 (ppm)

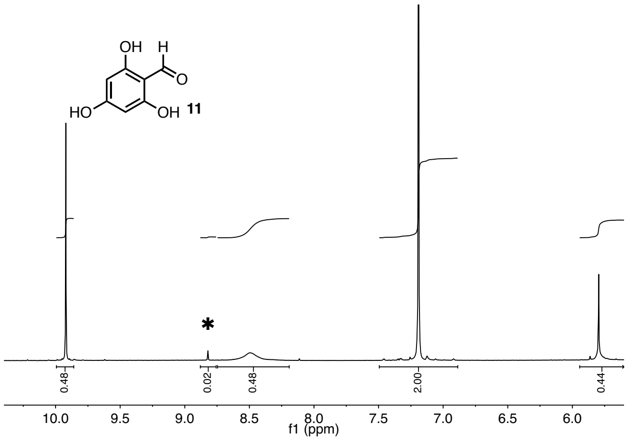
11
0.48
0.02
0.48
2.00
0.44
f1 (ppm)

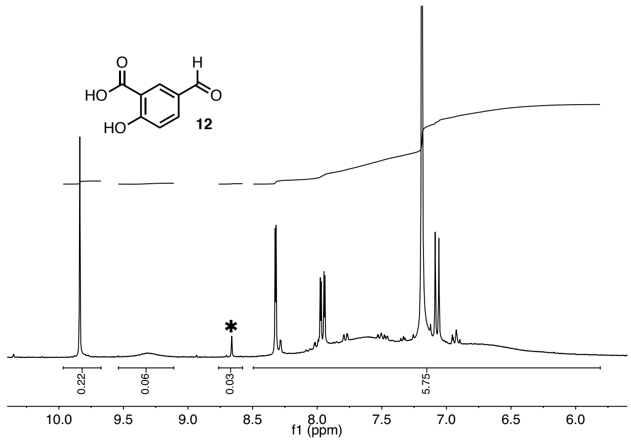
12
0.22
0.06
0.03
5.75
f1 (ppm)

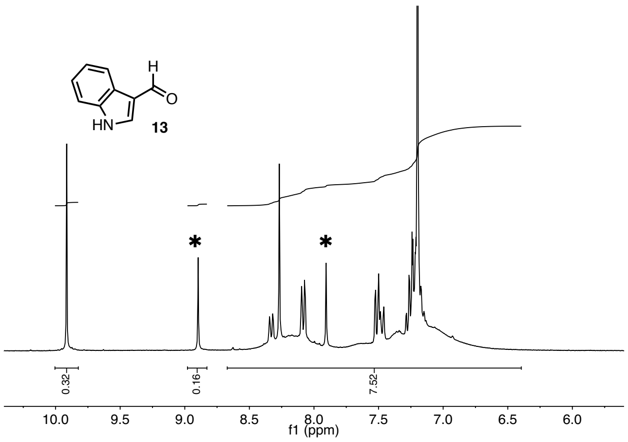
13
0.32
0.16
7.52
f1 (ppm)

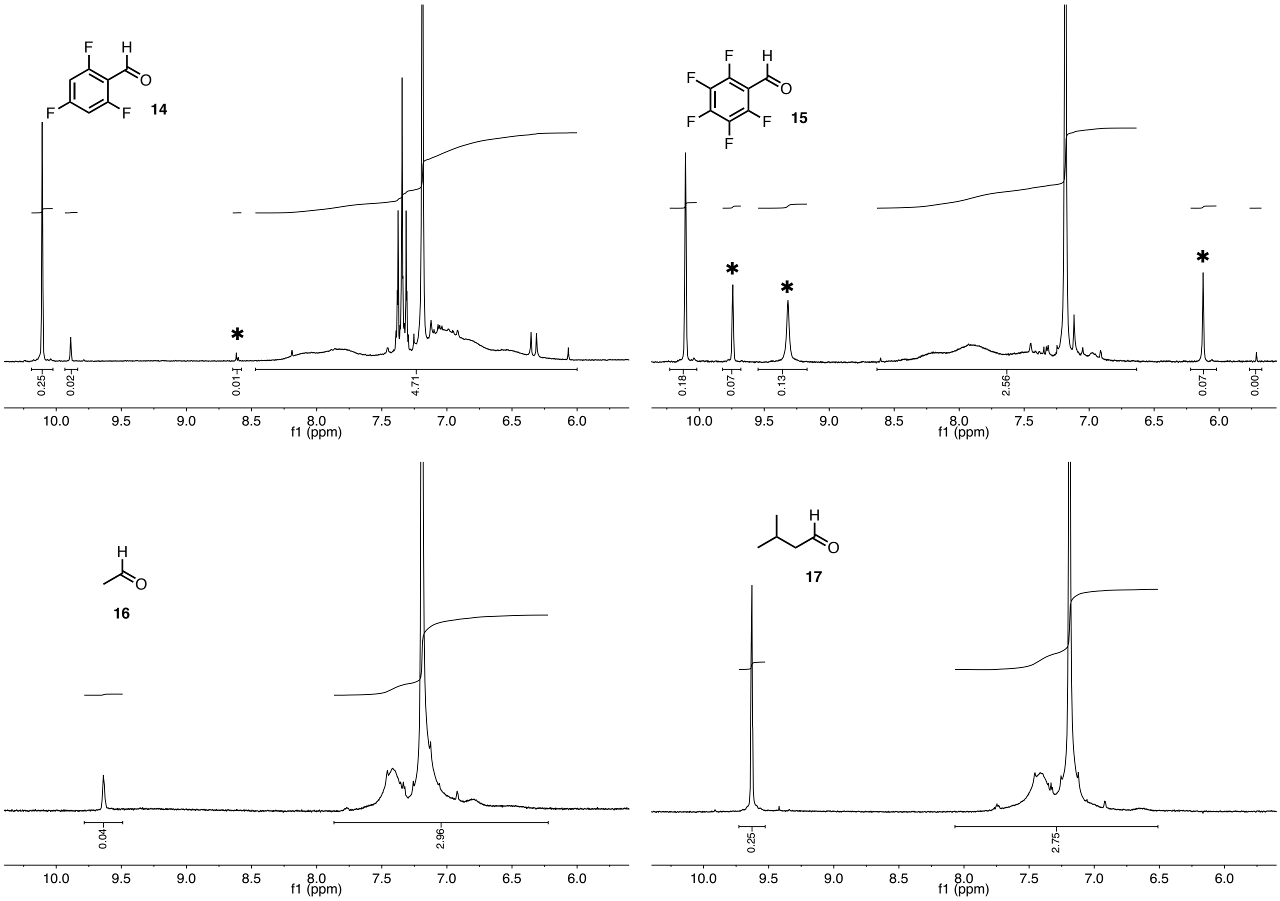

Fig. S15 Representative ^{1}H NMR spectra for the coupling of $\mathbf{P_{40}}$ with 1 eq. of different aldehydes. The sample were spiked with equimolar amounts of syringic acid. The integration values of the aromatic syringic acid signals (7.20 ppm) and the aromatic signals for the free and conjugated aldehydes were compared to the free aldehyde signal. *denotes the N-N*H*R impurity.

Table S2 Percentage loading in coupling reactions of $\mathbf{P_{40}}$ with selected aldehydes.

Entry	Aldehyde	Loading 1 eq.	Loading 2 eq.
$P_{40}1$	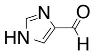	74%	**76%**
$P_{40}7$	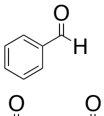	64%	**86%**
$P_{40}12$	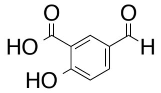	75%	**87%**
$P_{40}16$	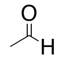	89%	**95%**
$P_{40}17$	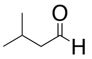	82%	**97%**

Reaction conditions: [$\mathbf{P_{40}}$]=77mM, 5% aqueous buffer (5% AcOH in D_2O) 95% DMSO-*d*6, t=24h, T=60 °C.

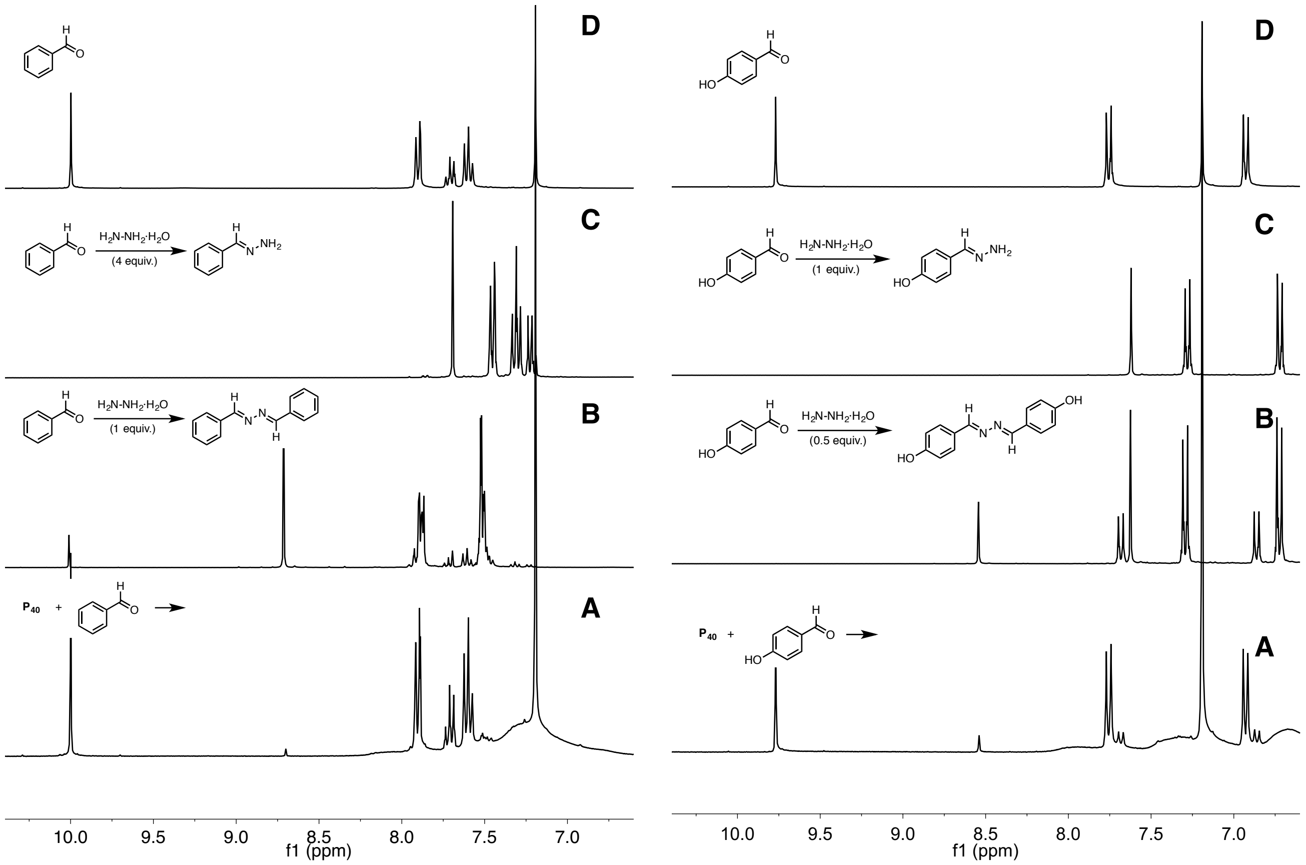

Fig. S16 Representative ^{1}H-NMR spectra of the reaction of $\mathbf{P_{40}}$ with 1 eq. of aldehyde (**A**), the reaction of the aldehyde with hydrazine monohydrate to afford mostly disubstituted hydrazine (**B**), the reaction of the aldehyde with hydrazine monohydrate to afford mostly monosubstituted hydrazine (**C**), and the aldehyde used (**D**). Left: benzaldehyde (**7**), Right: 4-hydroxybenzaldehyde (**9**). All samples were incubated in 95% DMSO-*d6* / 5% AcOH in D_2O.

Table S3 Percentage impurity in coupling reactions of $\mathbf{P_{40}}$ with 2 eq. of hydrophobic aldehydes.

Entry	Aldehyde	Impurity	Entry	Aldehyde	Impurity
$P_{40}7$	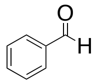	1%	$P_{40}14$	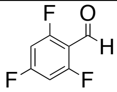	3%
$P_{40}8$	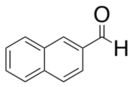	2%	$P_{40}15$	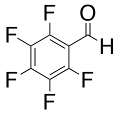	27%
$P_{40}9$	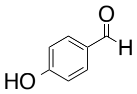	3%	$P_{40}16$	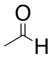	-
$P_{40}10$	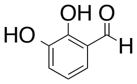	1%	$P_{40}17$	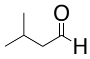	-
$P_{40}11$	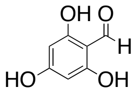	2%	$P_{40}18$	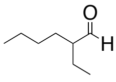	-
$P_{40}12$	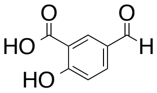	3%	$P_{40}19$	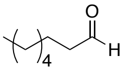	-
$P_{40}13$	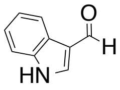	16%	$P_{40}20$	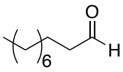	-

Reaction conditions: [$\mathbf{P_{40}}$]=77mM, 5% aqueous buffer (5% AcOH in D_2O) 95% DMSO-*d*6, t=24h, T=60 ^{o}C.

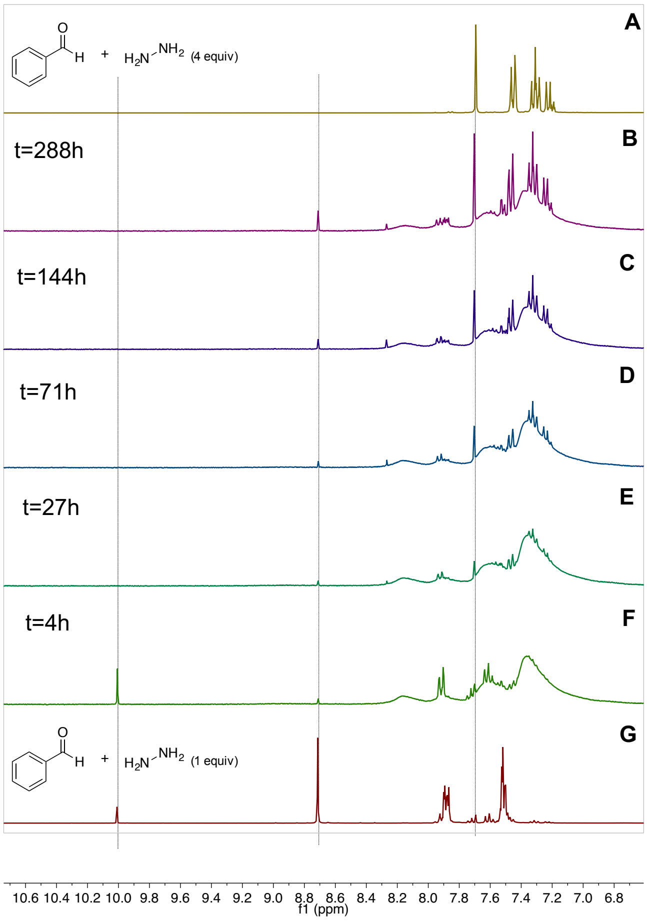

H

time / h	Aldehyde (10 ppm)	Monohydrazone (7.7 ppm)	Dihydrazone (8.7 ppm)	Total Impurities	In polymer
0	100.0%	0.0%	0.0%	0.0%	0.0%
1	32.9%	4.3%	0.5%	4.8%	62.3%
2	16.7%	2.4%	0.5%	3.0%	80.3%
3	8.1%	1.5%	0.7%	2.2%	89.7%
4	4.5%	1.0%	0.8%	1.8%	93.7%
27	0.0%	2.5%	0.7%	3.2%	96.8%
71	0.0%	7.0%	1.1%	8.1%	91.9%
96	0.0%	6.4%	1.1%	7.5%	92.5%
116	0.0%	7.9%	1.7%	9.6%	90.4%
144	0.0%	8.7%	1.8%	10.5%	89.5%
244	0.0%	13.2%	2.9%	16.1%	83.9%
288	0.0%	13.4%	3.7%	17.1%	82.9%

I

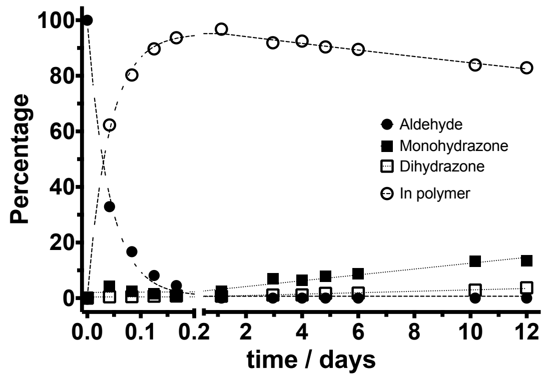

Fig. S17 ^{1}H-NMR spectra of the reaction of $\mathbf{P_{40}}$ reacted with 0.25 eq. of benzaldehyde (**7**) monitored at different intervals (**B-F**). ^{1}H-NMR spectra of the reaction of benzaldehyde (**7**) with 4 eq. (**A**) and 1 eq. (**G**) are shown for comparison.
(**H-I**) Relative changes in the intensity of the signal corresponding to aldehyde **7**, monohydrazone, dihydrazone and conjugated to the polymer as a function of time. The area for each peak was calculated using Mnova 8.1 line fitting tool. All samples were incubated in 95% DMSO-*d6* / 5% AcOH in D_2O.

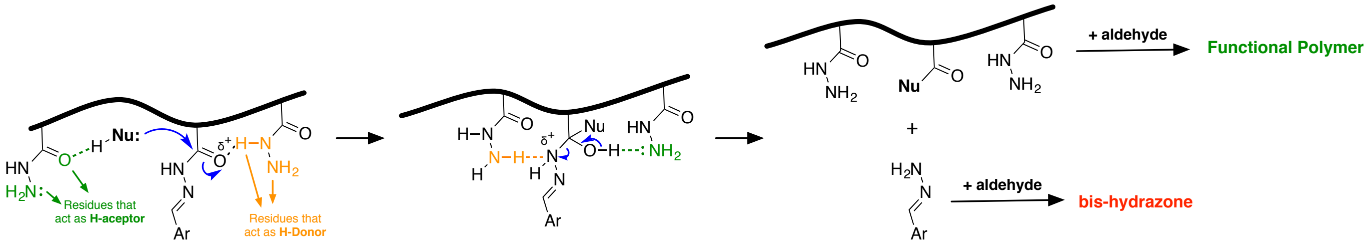

Scheme S1 Proposed mechanism for the formation of the impurities observed in the presence of aromatic aldehydes.

Table S4 Percentage loading and impurity in coupling reactions of $\mathbf{P_{80}}$ with selected aldehydes.

Entry	Aldehyde	Loading	Impurity	Entry	Aldehyde	Loading	Impurity
P_{80}1	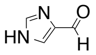	60%	1%	P_{80}12	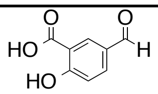	72%	2%
P_{80}7	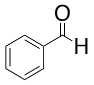	66%	1%	P_{80}13	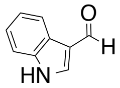	46%	12%
P_{80}8	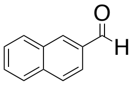	50%	1%	P_{80}16	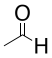	65%	-
P_{80}9	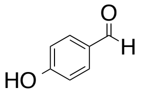	59%	7%	P_{80}17	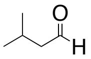	78%	-
P_{80}10	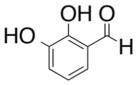	62%	1%				

Reaction conditions: [$\mathbf{P_{80}}$]=77mM, 5% aqueous buffer (5% AcOH in D_2O) 95% DMSO-*d*6, t=24h, T=60 ^{o}C.
